# Magnetization switching by combining electric field and spin-transfer torque effects in a perpendicular magnetic tunnel junction

**DOI:** 10.1038/srep18719

**Published:** 2016-01-06

**Authors:** Xiangli Zhang, Chengjie Wang, Yaowen Liu, Zongzhi Zhang, Q. Y. Jin, Chun-Gang Duan

**Affiliations:** 1 Department of Optical Science and Engineering, Key Laboratory of Micro and Nano Photonic Structures (Ministry of Education), Fudan University, Shanghai 200433, China; 2 School of Physical Science and Engineering, Tongji University, Shanghai 200092, China; 3 Key Laboratory of Polar Materials and Devices, Ministry of Education East China Normal University, Shanghai 200062, China

## Abstract

Effective manipulation of magnetization orientation driven by electric field in a perpendicularly magnetized tunnel junction introduces technologically relevant possibility for developing low power magnetic memories. However, the bipolar orientation characteristic of toggle-like magnetization switching possesses intrinsic difficulties for practical applications. By including both the in-plane (**T**_//_) and field-like (**T**_⊥_) spin-transfer torque terms in the Landau-Lifshitz-Gilbert simulation, reliable and deterministic magnetization reversal can be achieved at a significantly reduced current density of 5×10^9^ A/m^2^ under the co-action of electric field and spin-polarized current, provided that the electric-field pulse duration exceeds a certain critical value *τ*_c_. The required critical *τ*_c_ decreases with the increase of **T**_⊥_ strength because stronger **T**_⊥_ can make the finally stabilized out-of-plane component of magnetization stay in a larger negative value. The power consumption for such kind of deterministic magnetization switching is found to be two orders of magnitude lower than that of the switching driven by current only.

The effective manipulation of magnetization direction by electric current or voltage has attracted much attention for potential applications in nonvolatile spintronic devices. To date, different types of electric control methods have been developed: One is based on the spin-transfer torque (STT) effect induced by spin-polarized current^1^,^2^, another utilizes the voltage-controlled electric field (*E*-field) effect^3^, and the third is to use spin-orbit torques arising from either the spin-Hall effect^4^ or Rashba effect^5^,^6^. From a technological point of view, magnetization switching driven by these electric control methods is of great importance because reliable addressing of a single element bit inside dense arrays is much easier than that by magnetic field^7^. During the past fifteen years, investigations of current-driven magnetization reversal in spin valves^8^,^9^,^10^,^11^ or magnetic tunnel junctions (MTJs)^12^,^13^,^14^,^15^ have offered great promise for successful adaptation of the STT switching as a writing scheme in the advanced magnetic random access memory (STT-MRAM)^16^,^17^,^18^,^19^. However, compatibility of MTJ with the underlying transistor requires a critical switching current (*J*_c_) as low as ~10^9^ A/m^2^, which becomes the most challenging issue of high density memories. Meanwhile, reducing the STT switching current is also of particular importance to lower the energy consumption^19^,^20^. The magnetic memory devices with perpendicular magnetic anisotropy (PMA) were demonstrated to have great advantages over the in-plane ones, including strong thermal stability, low critical switching current density, and no restriction of shape aspect ratio^20^,^21^,^22^. Nevertheless, in spite of the great efforts made to decrease *J*_c_ , the typical *J*_c_ of the present memory devices is still of the order of 10^10^–10^11^ A/m^2^. Accordingly, new switching schemes are strongly expected for further reduction of power consumption.

In order to reduce energy consumption, magnetization switching driven by either spin-orbit torques^4^,^5^,^6^ or *E*-field effect^23^,^24^,^25^,^26^,^27^,^28^,^29^,^30^ has gained growing attention recently. In this study, we will focus on the *E*-field switching scheme. For the Fe/MgO or FePt/MgO-based perpendicular tunnel junctions, the application of electric field alters the Fermi level position at the interface, which changes the orbital occupancy and interface magnetic anisotropy^30^,^31^,^32^,^33^,^34^. The change of magnetic anisotropy will decrease the energy barrier of MTJs and lower the required critical energy for magnetization switching. Besides, the current-induced switching inevitably involves Joule heating, whereas the *E*-field control of magnetism in principle does not^30^. It has been predicted that the energy dissipation of magnetization switching with the assistance of *E*-field can be reduced by a factor of 500 when compared with the STT devices^27^. So far, two switching schemes regarding the *E*-field have been experimentally demonstrated in MTJs: one utilizes the coercivity change to assist magnetization reversal of the free layer^26^,^35^, while the other employs the temporal change of magnetic anisotropy to help magnetization switching in a “toggle-like” way^27^,^36^,^37^,^38^. Depending on the *E*-field pulse strength and duration (τ), the final magnetization state of free layer in the latter scheme case can be aligned either antiparallel (AP) or parallel (P) with the magnetization of fixed layer^37^,^38^,^39^. Compared to the STT switching, our previous study has predicted that ultrafast magnetization switching can be achieved by applying an *E*-field pulse as short as tens of picoseconds^38^. The significant reduction of switching time is attributed to the transient reduction in energy barrier, which saves the incubation switching time and consequently reduces the power consumption for a switching event^28^. However, the toggle-like switching has an unexpected feature that the final state strongly depends on the periodicity of magnetization precession, which leads to the indeterminacy in switching event due to difficulty in precise control over the pulse duration.

Undoubtedly, it is essential to achieve reliable deterministic magnetization switching with low power consumption for advanced perpendicular STT-MRAMs. One promising approach is to employ both the STT effect and *E*-field effect^40^,^41^. By this way, the deterministic magnetization switching is expected to be realized by the STT effect whilst the power consumption can be reduced with the *E*-field effect. In this paper, we performed simulations to reveal the magnetization switching behaviors driven by the combined action of electric field and STT effects. We find deterministic switching occurring at a much weaker current density but a larger pulse duration region which varies with the strength of field-like STT. The parameters of *E*-field and STT corresponding to such deterministic switching could be used for a new switching scheme with a dramatic reduction in energy consumption.

## Model

**Figure 1:**
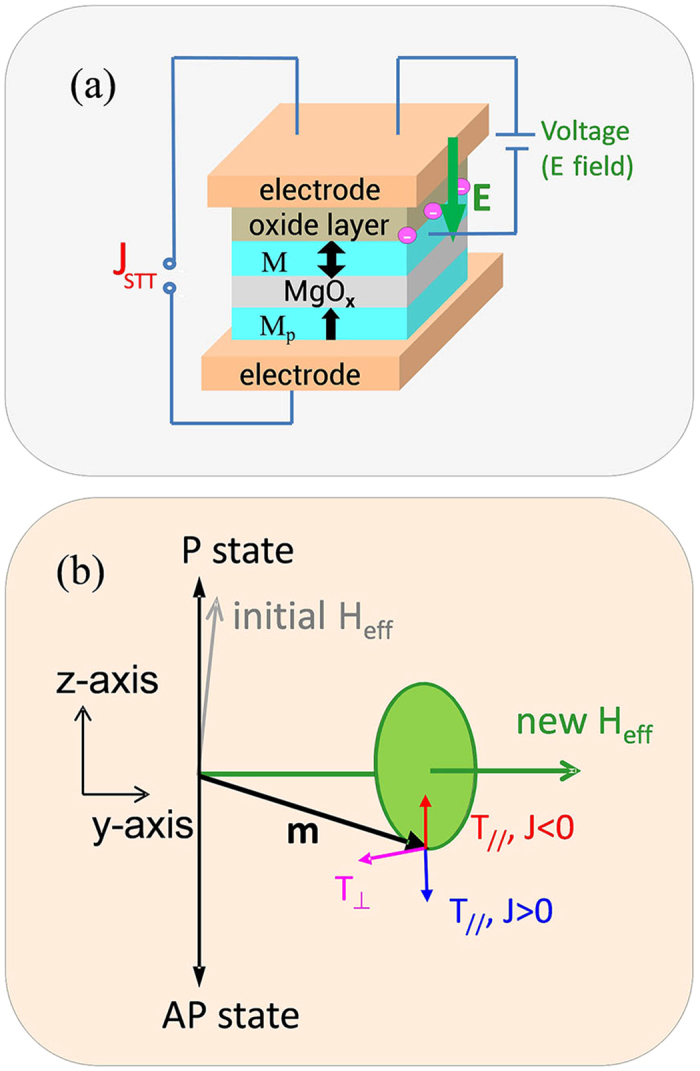
The electric field and spin-transfer torque effects on magnetization switching in a perpendicularly magnetized tunnel junction. (**a**) The schematic illustration of a magnetic tunnel junction under the action of electric field and spin-polarized current. (**b**) The orientations of free layer magnetization and the two components of spin transfer torque. The effective magnetic field direction with or without *E*-field pulse is also indicated.

The model structure we considered is a MgO/CoFeB-based perpendicularly magnetized tunnel junction. As illustrated in Fig. 1(a), a voltage (*E*-field) pulse is applied between the top electrode and the magnetic free layer separated by an oxide layer for tuning the PMA of free layer. The electric current is polarized by the fixed bottom ferromagnetic layer which exerts a spin-transfer torque on the free layer. We define the positive current as the electrons flowing from the free layer to the fixed layer. In our simulations, for the case of co-action of current and *E*-field pulses, the pulse length of injected current is 1 ns longer than the corresponding *E*-field pulse. Numerous studies have shown that the current-induced spin-transfer torque includes two vector components: the in-plane torque term^1^,^2^ and out-of-plane term (namely, the field-like torque)^42^,^43^. The field-like torque, with a strength as large as 10–30% of the in-plane torque, has a significant impact on the magnetization switching dynamics of MTJs^44^,^45^,^46^,^47^,^48^. It has been predicted the field-like STT can speed up the switching time and reduce the critical switching current^49^,^50^,^51^. Therefore, in this study, we simulated the magnetization dynamics by using the Landau-Lifshitz-Gilbert (LLG) equation which includes both the in-plane spin torque, 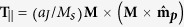 and the field-like spin torque, 
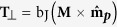
. Here 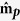 is the unit magnetization vector of the polarizer layer, *a*_*J*_ and *b*_*j*_ are the torque factors (see Methods). The typical material parameters are used for the CoFeB free layer^37^: free layer saturation magnetization μ_0_*M*_s_ = 1.4 T, Gilbert damping factor *α* = 0.2, thickness *d* = 1.4 nm, and spin polarization *P* = 0.3.

In this study, we suppose that an external field *H*_ext_ is applied with its out-of-plane component to completely compensate the stray field generated from the polarizer layer, and its in-plane component is denoted as *H*_y_ (along y-axis direction). As we know, under the action of voltage pulse, the effective magnetic field *H*_eff_ will change due to the reduced PMA. We suppose that the magnetic anisotropy field 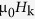 drops from 50 mT to −2 mT for the voltage increasing from zero to 1.8 V (i.e. *E* = 1.28 V/nm)^37^. That is to say, as a voltage of 1.8 V is applied, the effective magnetic field direction will vary from the initial z-axis to y-axis, as illustrated in Fig. 1(b). In this case, the free layer magnetization will precess around the new direction of effective magnetic field during the switching process.

## Results

**Figure 2:**
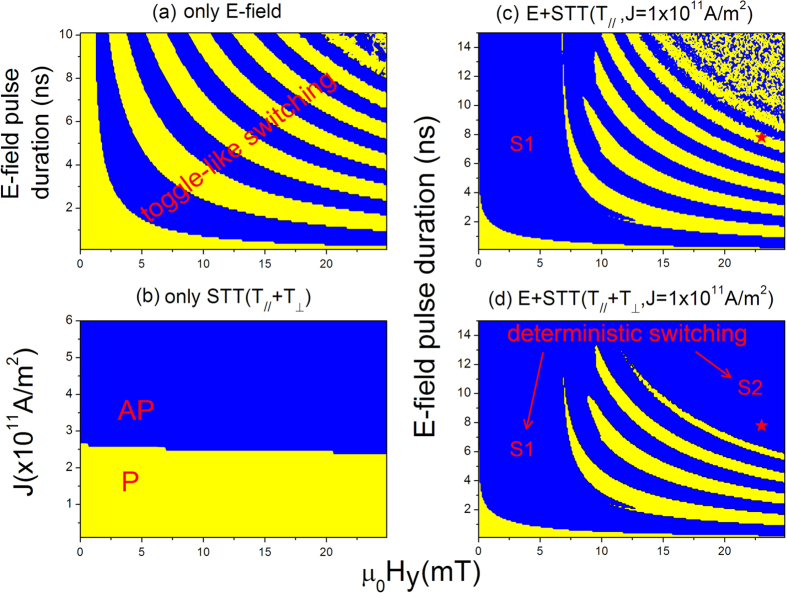
Phase diagram of the final magnetization state as a function of the *E*-field pulse, current density and magnetic field. (**a**) Final state driven by *E*-field effect alone for P→AP switching and (**b**) by STT effect alone with both the in-plane and field-like torques. (**c,d**) corresponds to the combined STT and *E*-field effects at 1.0×10^11^ A/m^2^ current without and with the field-like torque, respectively. Red stars represent the parameters used in Fig. 3.

We have carried out simulations on the magnetization switching driven by *E*-field only, STT only, or both *E*-field and STT. All the simulations start from an initial parallel configuration. Figure 2(a) shows the *E*-field induced magnetization switching phase diagram, for which a voltage pulse of 1.8 V with various pulse durations were used. As we mentioned, since the direction of effective magnetic field *H*_eff_ moves from z-axis to y-axis due to the PMA reduction caused by *E*-field, the magnetization will precess around the new *H*_eff_ direction [see Fig. 1(b)]. Once the voltage pulse vanishes, the magnetization vector of free layer, locating somewhere in a precession cycle, will ultimately relax towards one of the two equilibrium states: back to its initial P state (m_z_ = + 1) or switching to the AP state (m_z_ = −1). So, the final state of the free layer depends on the voltage pulse duration and external field, and this type of magnetization switching is termed as “toggle-like” switching mode^41^. The final P state (yellow stripes) and AP state (blue stripes) appears alternately in the parameter phase diagram. To reach an expected magnetization state, accurate parameter control (*E*-field pulse width and external field) is indispensable which holds a great challenge for experiments.

Reliable and deterministic switching is vitally important for practical applications, which can be realized by applying an enough strong current through the STT effect. Figure 2(b) shows the final magnetization states as functions of the applied current density *J* and in-plane external field *H*_y_ . The current pulse duration is set as 15 ns. Here both the in-plane and field-like spin-transfer torques are considered. In the simulation *ξ* is chosen to be 0.02, which is proportional to the strength of the field-like torque. Clearly, a critical current *J*_*c*_ as high as 2.5×10^11^ A/m^2^, which changes slightly with the external field, is required to achieve magnetization reversal from P to AP state. Obviously, the P-to-AP switching cannot occur if the current is below *J*_*c*_, for instance, *J* = 1×10^11^ A/m^2^, because the driving force by the STT alone with such low current cannot provide enough power to gyrate the free layer crossing over the energy barrier to another energetically favorite state. However, for the same low current density the P-to-AP switching can be achieved with the help of an additional *E*-field generated by a 1.8 V voltage pulse, as shown in Fig. 2(c,d). Interestingly, under the co-action of *E*-field and current, the parameter region of the first blue-stripe (AP state) is enlarged for both cases with [Fig. 2(c)] or without [Fig. 2(d)] the field-like component (**T**_⊥_) of STT, implying the expected deterministic AP state can be easily realized in this parameter region. This controllable AP region can be attributed to the STT effect which favors the AP state and dominates the magnetization dynamics^18^, referred to as “S1” switching region. More interestingly, for the STT term including **T**_⊥_, another even larger deterministic AP region appears at the top-right corner of Fig. 2(d), marked as “S2” switching region, suggesting that the field-like STT effect plays a significant role in enhancing the reliable magnetization switching of free layer. We would like to point out that similar features have also been observed for the AP to P switching at negative current case (not shown).

**Figure 3:**
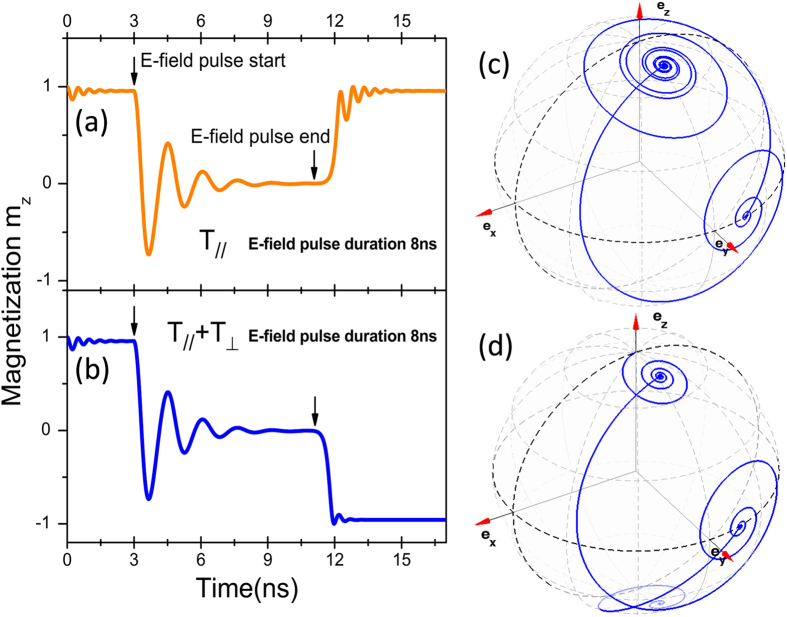
Magnetization dynamics driven by the combined STT and *E*-field effects. (**a,b**) Temporal dynamic processes of magnetization switching driven by STT without and with the **T**_⊥_ torque, respectively, at the same current density of 1.0×10^11^ A/m^2^. The in-plane component of the external field is 23 mT and the *E*-field pulse duration is 8 ns. (**c**,**d**) are the magnetization trajectories, which correspond to (**a,b**), respectively.

In order to distinguish the roles of the two spin torque terms of **T**_//_ and **T**_⊥_ on magnetization switching, we show in Fig. 3 the temporal evolutions of free layer magnetization simulated under the same *E*-field and current. The only difference is that only the in-plane **T**_//_ is included in Fig. 3(a,c) while Fig. 3(b,d) contains both the **T**_//_ and **T**_⊥_ torques. The simulation parameters are taken as *H*_y_ = 23 mT and τ = 8 ns (1.8 V in strength), corresponding to the parameter points marked as the red stars in Fig. 2(c,d). First we applied a current density of 1.0×10^11^ A/m^2^ to generate the STT effect. Clearly, the STT alone in both cases cannot cause the magnetization to deviate away from its initial equilibrium state since such low current is far from enough to switch the magnetization [see Fig. 2(b)]. At *t* = 3 ns, the *E*-field pulse is added, which modifies the effective field, leading to the magnetization of free layer away from the initial +*z* direction and precessing around the in-plane +*y* direction [see Fig. 3(c,d)]. As soon as the *E*-field is removed, no switching takes place and the free layer magnetization returns back to the P state if the field-like toque is not considered. On the contrary, the magnetization switches to the -*z* direction (AP state) when both **T**_//_ and **T**_⊥_ torques are included in the simulation model.

**Figure 4:**
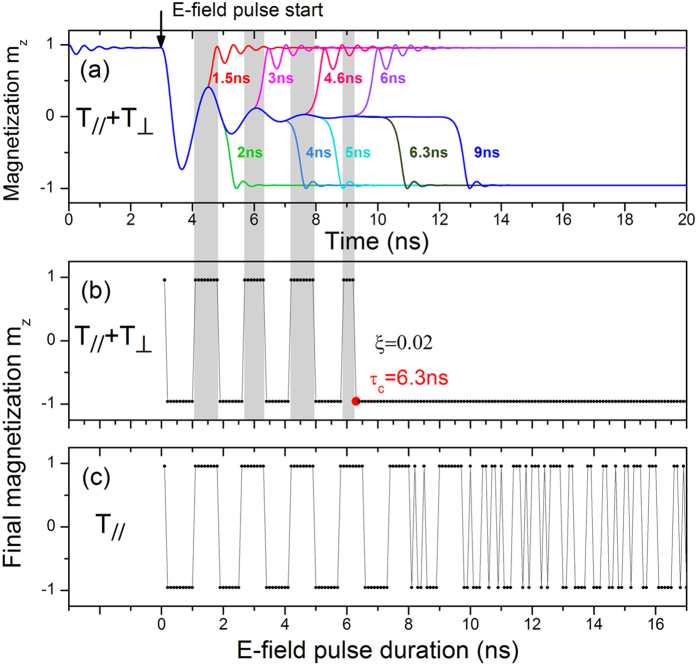
The dependence of magnetization state on the pulse duration of electric field. (**a**) Temporal processes of magnetization switching driven by the combined effects at various pulse durations with both the **T**_//_ and **T**_⊥_ torques. The initial state is the parallel state and the electric field pulse begins at t = 3 ns. The gray shadow area represents the non-switching zone when the electric pulse ends. The perpendicular component of the final magnetization as a function of *E*-field pulse duration after applying both the **T**_//_ and **T**_⊥_ torques (**b**) or only the **T**_//_ torque (**c**). *τ*_*c*_ is the critical value of pulse duration for deterministic switching.

To further clarify that, we have performed a series of simulations using the same parameters including the current density of 1.0×10^11^ A/m^2^, the external field of *H*_y_ = 23 mT, and the voltage of 1.8 V, but different *E*-field pulse durations. Figure 4(a) shows the magnetization evolutions after considering both the **T**_//_ and **T**_⊥_ torques. We can see that the free layer magnetization goes back to the parallel configuration for the pulse duration τ = 1.5, 3.0, 4.6 and 6.0 ns, whereas it switches to the AP configuration for τ = 2, 4, 5, and 6.3 ns, respectively. This is a typical toggle-like switching mode. However, as the *E*-field pulse is longer than 6.3 ns, the final AP state can be observed only, thus producing a deterministic magnetization switching region. This feature has been further confirmed by continuously changing the pulse duration from 0 to 30 ns. The dependence of final magnetization state on the pulse duration is given in Fig. 4(b) (here we just show the results for τ < 17 ns). Clearly, we can see that deterministic switching will occur as long as the pulse duration exceeds 6.3 ns, which means the free layer will be certainly switched from P state to AP state. As a result, we consider here the critical pulse duration *τ*_c_ of *E*-field is 6.3 ns. For comparison, we have also performed simulations with the in-plane torque only. As shown in Fig. 4(c), the final state always changes periodically between the AP state and P state for the *E*-field pulse duration smaller than 7.8 ns, above which random magnetization switching is observed. This means that the in-plane torque alone cannot generate deterministic switching even though the *E*-field pulse duration is long enough to stabilize the magnetization along the in-plane direction.

**Figure 5:**
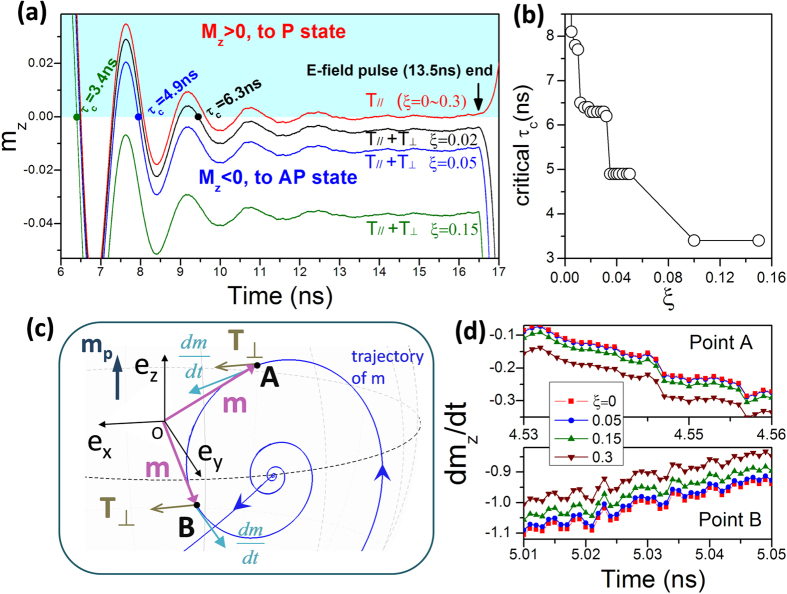
Influence of field-like STT on the pulse duration of electric field to trigger the deterministic magnetization switching. (**a**) The temporal evolution of the z-component of magnetization near the in-plane equilibrium position during the *E*-field action for various field-like torque strengths of *ξ* = 0, 0.02, 0.05, and 0.15. The cyan shadow area represents the positive m_z_. (**b**) The critical pulse duration *τ*_*c*_ for deterministic switching is plotted as a function of *ξ*. (**c**) Schematic of magnetization trajectory around the in-plane equilibrium position and the field-like torque involved in the influence of d**m**/dt. The magnetization trajectory is indicated by the blue curve. The torque **T**_⊥_, magnetization **m**, and d**m**/dt are represented by brown, purple and cyan arrows, respectively. (**d**) The z-component of d**m**/dt at point A and point B for various *ξ*.

The required critical pulse duration *τ*_c_ to trigger the deterministic magnetization switching is found to be closely related to the strength of field-like STT. Figure 5(a) shows the zoom-in magnetization evolutions for different **T**_⊥_ strengths characterized by a parameter *ξ*. In these simulations, the *E*-field pulse duration is fixed to be 13.5 ns and the other parameters are the same as those in Fig. 4. Note that, for the case of **T**_//_ torque only, the out-of-plane magnetization component *m*_z_ exhibits damped precession which finally stabilizes around the *m*_z_ = 0 axis during the electric pulse duration. The final P or AP state is dominated by the fluctuated *m*_z_ value at the moment when the *E*-field pulse is just finished, which can be either positive or negative depending on the pulse duration but not on the *ξ* strength (*ξ* changes from 0 to 0.3). For the positive *m*_z_ [stay in the cyan region, see Fig. 5(a)], the recovered strong PMA drives the free layer magnetization to the P state, whilst for the negative *m*_z_ the PMA forces it to the final AP state. This result is responsible for the observed random switching state shown in Fig. 4(c), implying that the influence of **T**_//_ on the new deterministic switching region is very weak and can be ignored. However, when the **T**_⊥_ component is included, the *m*_z_ value will be shifted downwards and keeps negative when the pulse duration is over a critical value, e.g. *τ*_c_ = 6.3 ns for *ξ* = 0.02. Such shift makes the *m*_z_ stabilize at a negative value, which would certainly result in a deterministic AP magnetization switching as the pulse is removed. As a result, from the oscillation curves shown in Fig. 5(a), we can easily obtain the critical pulse duration *τ*_c_, over which the *m*_z_ stays negative and AP state occurs. The *τ*_c_ value relies on the *ξ*, which decreases down to 4.9 and 3.4 ns as *ξ* increasing up to 0.05 and 0.15, respectively. For clarity, the critical pulse durations are summarized in Fig. 5(b) as a function of the field-like torque parameter *ξ*.

The effect of field-like torque on the stabilized *m*_z_, i.e. the equilibrium position of *m*_z_ during the action of *E*-field, can be qualitatively analysed from the LLG equation governed by equation (1). Figure 5(c) shows the zoom-in precession trajectory of Fig. 3(d) near the equilibrium position for *ξ* = 0.02. In this case since the precession vector **m** is close to the y-axis, the direction of **T**_⊥_ is approximately along +*x* direction. The directions of **T**_⊥_ (brown arrows) and 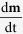 (cyan arrows) are indicated for two typical points (A and B). At point “A” (somewhere in the region of *m*_z_ > 0), the **T**_⊥_ has a component parallel to 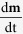, leading to an increase in the amplitude of dm_z_**/**dt which is proportional to the factor *ξ*, as shown in Fig. 5(d). As a result, the damping term, in a form of 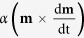 will increase as well with **T**_⊥_, which can reduce the radius of precession, i.e. the oscillation amplitude of *m*_z_, being in agreement with the results shown in Fig. 5(a). In contrast, at point “B” (somewhere of *m*_z_ < 0), because the **T**_⊥_ has a component antiparallel to 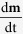, the oscillation amplitude of *m*_z_ gets larger with increasing *ξ* due to the partially canceled damping term. Consequently, in the presence of **T**_⊥_, the *m*_z_ oscillation curves are shifted downwards, which eventually stabilizes at a negative value during the *E*-field pulse. Therefore, deterministic magnetization switching to the antiparallel state (*m*_z_ = −1) can be achieved if the pulse duration is larger than *τ*_c_.

**Figure 6:**
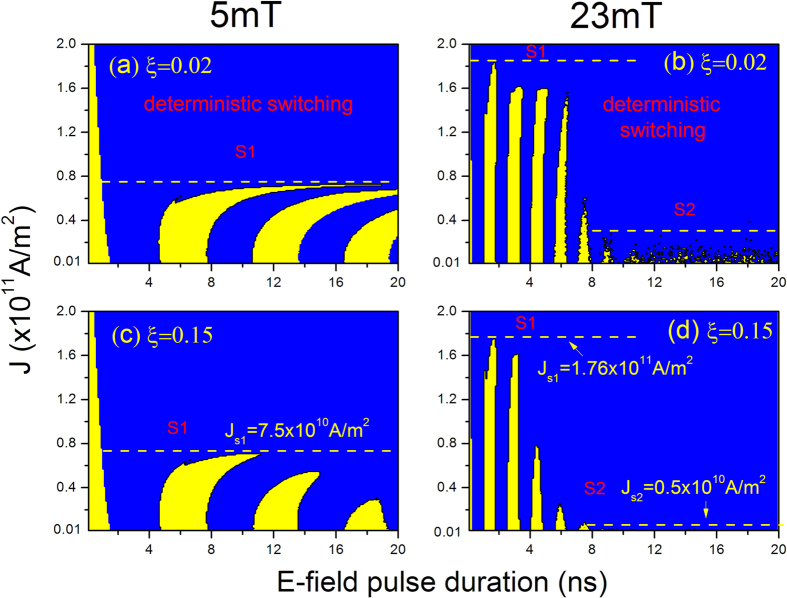
The dependence of toggle-like and deterministic magnetization switching on the applied current and *E*-field pulse. The simulations are performed with the co-action of the STT and *E*-field effects at a field of *H*_y_ = 5 mT (left panel) or 23 mT (right panel), where *ξ* is chosen as 0.02 and 0.15.

Figure 6 shows the phase diagrams of the final magnetization states of the free layer under different current densities (STT effect) and *E*-field pulse durations at a field of *H*_y_ = 5 mT (left panel) or 23 mT (right panel), where *ξ* is chosen as 0.02 and 0.15. Clearly, with the combined effects, the critical switching current is significantly reduced. The magnetization switching can be achieved by two different switching modes: toggle-like switching (the alternate blue-yellow stripes) at the case of shorter *E*-field pulse and weaker current density, and deterministic switching (the continuous blue area) for the *E*-field pulse longer than a critical value in “S2” switching region or current density higher than a critical value in “S1” switching region (such a value is still smaller than the critical current without the *E*-field effect, see Fig. 2(b)). This result is in qualitative agreement with the experimental results of ref. 40in which the switching is achieved by applying a pulse for *E*-field effect followed by a pulse for STT effect. Note that, in the case of H_y_ = 5 mT, we only observe the S1 switching region during the 20 ns *E*-field pulse duration for both *ξ* = 0.02 and 0.15 [Fig. 6(a,c)], while at H_y_ = 23 mT case we can see both the S1 and S2 switching region [Fig. 6(b,d)]. Obviously, the magnetization switching in the S1 region requires much larger current (7.5×10^10^ A/m^2^ at H_y_ = 5 mT or 1.76×10^11^ A/m^2^ at H_y_ = 23 mT) although the *E*-field pulse duration can be reduced down to 2 ns. In contrast, in the S2 region, the reliable switching can be achieved at a current density of 0.5×10^10^ A/m^2^ and an *E*-field pulse of 8 ns [see Fig. 6(d)]. Additionally, one can notice that in the S2 switching region the enhanced field-like torque via a larger *ξ* can reduce the critical pulse duration *τ*_c_, which has been observed in Fig. 5(b).

For practical applications, the power cost for driving the magnetization switching is a key factor to be considered. The required STT power is calculated according to 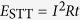 and the *E*-field power is estimated by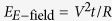, where *I* is the current for STT, *V* is the voltage for generating *E*-field pulse, *R* is the resistance of MTJ, *t* is the function time of STT or *E*-field. We use the typical experimental parameters for MgO-based MTJ^37^: 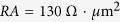 and D = 70 nm (diameter). Here we mainly focus on the deterministic switching in S1 and S2 regions because it is more easily to be controlled in experiments. In S1 region of Fig. 6(c), if we choose a short *E*-pulse of *τ *= 2 ns and a switching current of 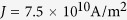 with a 3 ns current pulse time, the respective energy consumption is 8.4 pJ for STT effect and 0.19 pJ for *E*-field effect. In S2 region of Fig. 6(d), if we take the *E*-field pulse as *τ *= 8 ns, a current density 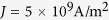 and a current pulse duration 10 ns, the energy consumption is estimated as 0.13 pJ consumed by the STT effect and 0.77 pJ by the *E*-field pulse. The total energy consumption in both cases are much smaller than the switching driven by STT effect alone, which is around 312 pJ for 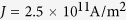 and a switching time of 10 ns. Obviously, compared with the STT alone writing scheme, the power cost can be reduced by a factor of 37 for the deterministic switching in the S1 region while by a factor of 347 in the S2 region. Note that in these calculations, the power cost for generating the magnetic field was neglected.

In summary, we have investigated magnetization switching properties in MgO-based magnetic tunnel junctions driven by the electric-field effect and spin-transfer torque effect. The magnetization switching of free layer driven by *E*-field only is toggle-like, with final state either parallel or antiparallel with the reference layer, depending on the *E*-field pulse duration and external magnetic field. In contrast, reliable and deterministic switching can be realized by STT only under a quite strong current, which will limit the practical applications. By using a new switching scheme combining both the *E*-field pulse and STT current, the power cost for deterministic magnetization switching could be significantly reduced by two orders of magnitude if both the in-plane and field-like torques are considered. The deterministic switching can occur at a reduced current density of 10^9^ A/m^2^ as the *E*-field pulse duration is larger than a critical value, which is found to decrease with the increase of field-like STT term. The reliable and deterministic magnetization switching with low power consumption promises potential advantages for the future spintronic devices, especially for low power writing of the MRAMs.

## Methods

The roles of electric field and spin-polarized current acting on the free layer are modeled by including a change of perpendicular anisotropy and spin-transfer torque terms, respectively, into the Landau-Lifshitz-Gilbert (LLG) equation. In this study, macrospin simulations are carried out by solving the LLG equation, which is taken as:


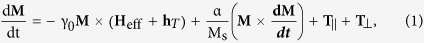


The simulation code is developed in-house and has been used in our previous study^38^,^41^. Here **M** = **m***M*_s_ is the magnetization vector of the free layer, **m** is the unit magnetization vector, *M*_s_ is the free layer saturation magnetization, ***H***_eff_ is the effective magnetic field that includes the external magnetic field, anisotropy field and demagnetization field. ***h** *_T_ is an instantaneous thermal fluctuation field and in this study we set the temperature T = 300 K. The third term is the in-plane spin torque, 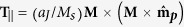, and the fourth term is the field-like spin torque, 
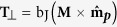
. Here 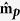 is the unit magnetization vector of the polarizer layer. The torque factors 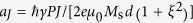 and 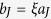, where *γ* is the gyromagnetic constant, 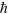 is the Planck constant, *J* is the electric current density, *d* is the free layer thickness, *e* is the electron charge, *P* is the spin polarization. 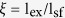, which denotes the ratio of spin decoherence length and spin-flip relaxation length^48^.

## Additional Information

**How to cite this article**: Zhang, X. *et al.* Magnetization switching by combining electric field and spin-transfer torque effects in a perpendicular magnetic tunnel junction. *Sci. Rep.* **6**, 18719; doi: 10.1038/srep18719 (2016).
